# The Effect of Ascorbic Acid and Nicotinamide on Panton–Valentine Leukocidin Cytotoxicity: An Ex Vivo Study

**DOI:** 10.3390/toxins15010038

**Published:** 2023-01-04

**Authors:** Abdullah AlSaleh, Mohammed Shahid, Eman Farid, Khalid Bindayna

**Affiliations:** Department of Microbiology, Immunology and Infectious Diseases, College of Medicine and Medical Sciences, Arabian Gulf University, Manama 329, Bahrain

**Keywords:** PVL, antioxidant, vitamin, ascorbic acid, nicotinamide

## Abstract

Background: Panton–Valentine Leukocidin sustains a strong cytotoxic activity, targeting immune cells and, consequently, perforating the plasma membrane and inducing cell death. The present study is aimed to examine the individual effect of ascorbic acid and nicotinamide on PVL cytotoxicity ex vivo, as well as their effect on granulocytes viability when treated with PVL. Materials and Methods: The PVL cytotoxicity assay was performed in triplicates using the commercial Cytotoxicity Detection Kit PLUS (LDH). LDH release was measured to determine cell damage and cell viability was measured via flow cytometry. Results and discussion: A clear reduction in PVL cytotoxicity was demonstrated (*p* < 0.001). Treatment with ascorbic acid at 5 mg/mL has shown a 3-fold reduction in PVL cytotoxicity; likewise, nicotinamide illustrated a 4-fold reduction in PVL cytotoxicity. Moreover, granulocytes’ viability after PVL treatment was maintained when incubated with 5 mg/mL of ascorbic acid and nicotinamide. Conclusions: our findings illustrated that ascorbic acid and nicotinamide exhibit an inhibitory effect on PVL cytotoxicity and promote cell viability, as the cytotoxic effect of the toxin is postulated to be neutralized by antioxidant incubation. Further investigations are needed to assess whether these antioxidants may be viable options in PVL cytotoxicity attenuation in PVL-associated diseases.

## 1. Introduction

Panton–Valentine Leukocidin (PVL) is a bi-component pore-forming staphylococcal exotoxin that has shown remarkable emergence attributable to being commonly harbored by epidemic community-associated staphylococcal strains [1]. It sustains a strong cytotoxic activity, targeting immune cells, mainly neutrophils, and to some extent, monocytes and macrophages, perforating the plasma membrane of these immune cells and inducing cell death [2,3]. PVL has been associated with complications in lung injuries, necrotizing pneumonia, skin infections, osteomyelitis, necrotizing fasciitis, and inflammatory cytokine storms [4,5,6,7].

The two components comprising PVL’s β-barrel configuration are the LukS subunit (slow) and the LukF subunit (fast). These components are released by the bacteria into the microenvironment as un-assembled monomers [8]. Initially, the S-subunit binds to the target cell membrane, which prompts the recruitment of the F-subunit; hence, oligomerization may ensue, resulting in the formation of a lytic hetero-octameric pore-forming complex [8,9,10]. Moreover, the main immune cell receptor that mediates LukS-PV binding and PVL cytotoxicity is the C5a anaphylatoxin chemotactic receptor 1 (C5aR1); also, C5aR2 is targeted but with lesser affinity [11].

The mechanism by which PVL induces cell death is not fully understood; however, it has been elucidated that PVL activity induces potassium ions (K^+^) efflux and, in turn, calcium ions (Ca^2+^) influx, as well as cathepsin B (CTSB) maturation [12,13], activating NLRP3 inflammasome and leading to the secretion of pro-inflammatory cytokines, such as IL-1β [12,14]. In turn, IL-1β facilitates the upregulation of numerous proinflammatory components such as TNF, IL-6, Phospholipase A2 (PLA-2), the Intracellular Adhesion Molecule -1 (ICAM-1), and Type-2 Cyclooxygenase (COX-2), among others, thus perpetuating the inflammatory effect of PVL [15]. It is worth noting that cellular stress induced by PVL prompts granulocytes to release additional proinflammatory mediators, including leukotriene B4 (LTB4), IL-8, and histamine [16,17].

Despite the substantial epidemiological association between PVL and necrotizing infections, PVL pathophysiology is yet to be fully elucidated. One major hurdle in investigating PVL cytotoxicity is that it exhibits activity in a species-specific manner, as it shows affinity toward human neutrophils but not murine, macaque, or cow receptors [18]. However, few successful models have been developed. PVL-treated rabbit neutrophils have shown somewhat substantial cytotoxic activity, as have humanized mice models (non-obese diabetic (NOD)/severe combined immune deficiency (SCID)/IL2Rγnull (NSG) mice engrafted with primary human hematopoietic cells) in pneumonia and skin infection models [19,20,21].

Recently, antioxidants have gained notoriety for their antimicrobial and anti-inflammatory effects, whether by upregulating anti-inflammatory genes (e.g., β-carotene and lycopene), downregulating bacterial virulence genes (e.g., cholecalciferol and α-tocopherol), or improving the efficacy of current antimicrobial agents (e.g., ascorbic acid, nicotinamide, and α-tocopherol). Indeed, they may provide a viable option for supportive therapy [22,23,24,25,26,27]. In particular, ascorbic acid and nicotinamide may prove to be outstanding candidates. They are cheap, widely available, water-soluble, and have been used in clinical trials for their antimicrobial, anti-inflammatory, and antioxidant properties; henceforth, they provide a reliable option to be considered in this ex vivo experiment.

The aim of this study is to examine the individual effect of ascorbic acid and nicotinamide on PVL cytotoxicity ex vivo, as well as their effect on granulocyte viability when treated with PVL. To our knowledge, these interactions between PVL and vitamins B3 and C have never been investigated before. Nevertheless, we believe that antioxidants can, in fact, enhance the clinical picture of many ailments, with due future research.

## 2. Results and Discussion

Ascorbic acid, commonly known as vitamin C, is an essential water-soluble micronutrient that is involved in maintaining homeostatic body function, as well as in the absorption of iron, the biosynthesis of carnitine, catecholamines and collagen, and in preventing the development of dental caries [28,29,30]. It is found in fresh vegetables and fruits such as broccoli, tomatoes, strawberries, kiwi, mangoes, citrus fruits, and leafy vegetables [31]. Vitamin C is commonly described as an antioxidant that reduces reactive oxygen species (ROS) and free radicals; however, at high concentrations, it may exhibit prooxidant properties, generating ROS. So, the precise mechanism of action of vitamin C is still debatable [32,33,34].

Vitamin C demonstrated bactericidal and virulence-suppressing effects on hypervirulent *Klebsiella pneumoniae*, *Pseudomonas aeruginosa*, *Bacillus subtilis*, and *Escherichia coli* by actively suppressing the production of biofilm exopolysaccharides, as well as downregulating biofilm-associated genes and antibiotic resistance genes when treated with subinhibitory concentrations of ascorbic acid [25,34,35,36]. Additionally, vitamin C has been reported to synergize with antibiotics and improve their efficacy against gram-positive and gram-negative bacterial superbugs [27,37].

Nicotinamide is the amide water-soluble form of vitamin B3, manifesting as a precursor for the co-enzyme nicotinamide adenine dinucleotide (NAD^+^); thus, it is involved in thousands of metabolic pathways [38,39]. Meat, fish, corn, and wheat are the primary natural sources of nicotinamide, while vegetables and fruits may present lesser content [40].

It is a pellagra-preventing agent that demonstrates cytoprotective characteristics in degenerative and immunity-related diseases [41]. Oral supplementation of nicotinamide improved diastolic dysfunction and enhanced myocardial bioenergetics in rodent models [42]. In a clinical trial, nicotinamide mononucleotide supplements increased muscle insulin sensitivity in obese prediabetic women [43]. Moreover, vitamin B3 supplementation modulates neuroprotective roles in Alzheimer’s, Parkinson’s, and Huntington’s diseases, as well as downregulates proinflammatory cascades involving NLRP3 and ASC [44,45]. Nicotinamide also demonstrates virulence-suppressing effects against bacterial pathogens by actively restricting the intracellular growth of *Mycobacterium tuberculosis* in immune cells, as well as inhibiting biofilm formation and exopolysaccharides production in *streptococcus mutans* in a rat caries model [46,47].

**Figure 1:**
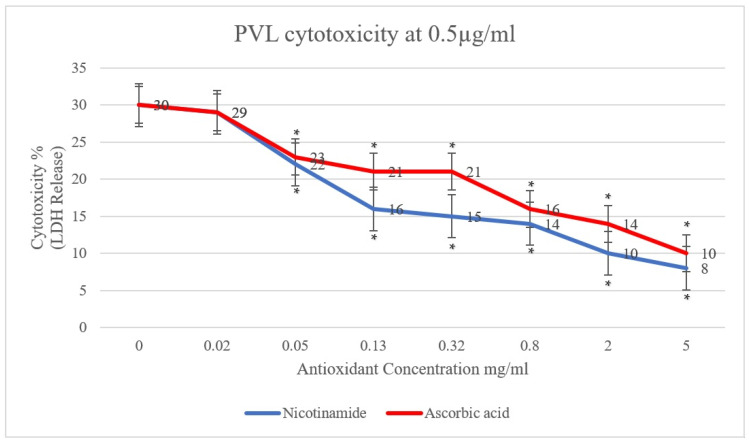
PVL (0.5 µg/mL) cytotoxicity rates were determined through LDH released from white blood cells (WBCs) via the commercial Cytotoxicity Detection Kit PLUS. WBCs were incubated with serial dilutions of ascorbic acid and nicotinamide (5, 2, 0.8, 0.32, 0.13, 0.05, and 0.02 mg/mL) individually. At 0 mg/mL, the assay included WBCs and PVL only. All assays were conducted in triplicates. A *t*-test was conducted to determine the statistical significance of antioxidant treatment when compared with the control (0 mg/mL). Statistical significance (*p* < 0.001) is represented by an asterisk.

**Figure 2:**
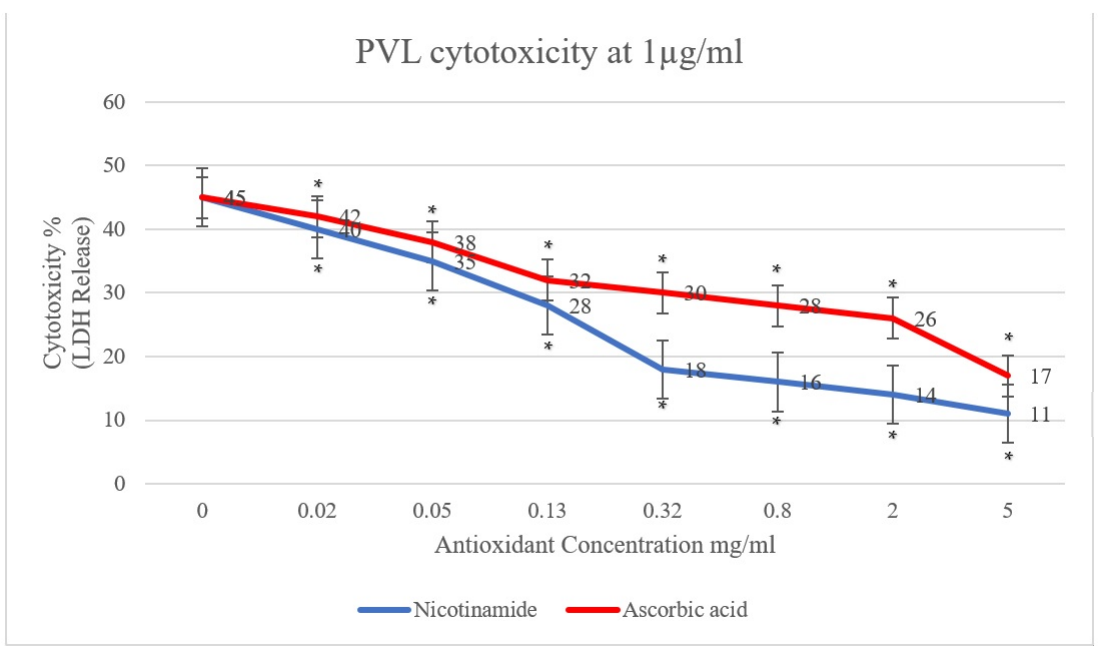
PVL (1 µg/mL) cytotoxicity rates determined through LDH released from white blood cells (WBCs) via the commercial Cytotoxicity Detection Kit PLUS. WBCs were incubated with serial dilutions of ascorbic acid and nicotinamide (5, 2, 0.8, 0.32, 0.13, 0.05, and 0.02 mg/mL) individually. At 0 mg/mL, the assay included WBCs and PVL only. All assays were conducted in triplicates. A *t*-test was conducted to determine the statistical significance of antioxidant treatment when compared with the control (0 mg/mL). Statistical significance (*p* < 0.001) is represented by an asterisk.

In the present study, the effect of antioxidants (ascorbic acid and nicotinamide) on PVL cytotoxicity was investigated. A clear reduction in PVL cytotoxicity was demonstrated, positively proportional to the concentration of ascorbic acid and nicotinamide individually (*p* < 0.001). As shown in Figure 1, PVL cytotoxicity at a toxin concentration of 0.5 µg/mL decreased from 30% to 10% and 8% when incubated with 5 mg/mL of ascorbic acid and nicotinamide, respectively (*p* < 0.001). Additionally, at a PVL concentration of 1 µg/mL (Figure 2), cytotoxicity decreased from 45% to 17% and 11% when incubated with 5 mg/mL of ascorbic acid and nicotinamide, respectively (*p* < 0.001). It is worth noting that the concentrations of ascorbic acid and nicotinamide used in this study represent a range of sub-inhibitory concentrations for PVL-producing *S. aureus*. Indeed, the reported minimum inhibitory concentration (MIC), *S. aureus* was 10–5 mg/mL and 60–30 mg/mL for ascorbic acid and nicotinamide, respectively [27].

The cytotoxic effect of PVL on granulocytes has been reported at concentrations as low as 5 ng/mL in vitro; in fact, neutrophil necrosis was induced with PVL treatment at a concentration of 0.04 µg/mL [16,19]. Additionally, PVL in clinical specimens was reported to range from 0.27 to 2 µg/mL when analyzing pus samples [48]. So, in this study, using two concentrations (0.5 and 1 µg/mL) in the mid-range of PVL concentrations in clinical specimens would provide a reasonable indication of the effectivity of the tested antioxidants. Furthermore, treatment with ascorbic acid at 5 mg/mL has shown a 3-fold consistent reduction in PVL cytotoxicity; likewise, 5 mg/mL of nicotinamide illustrated a 4-fold reduction in PVL cytotoxicity, as seen in Supplementary Tables S1 and S2. It is worth noting that a statistically significant reduction in PVL cytotoxicity with lower concentrations of the tested antioxidant has been demonstrated down to 0.05 mg/mL (Supplementary Tables S1 and S2).

**Figure 3:**
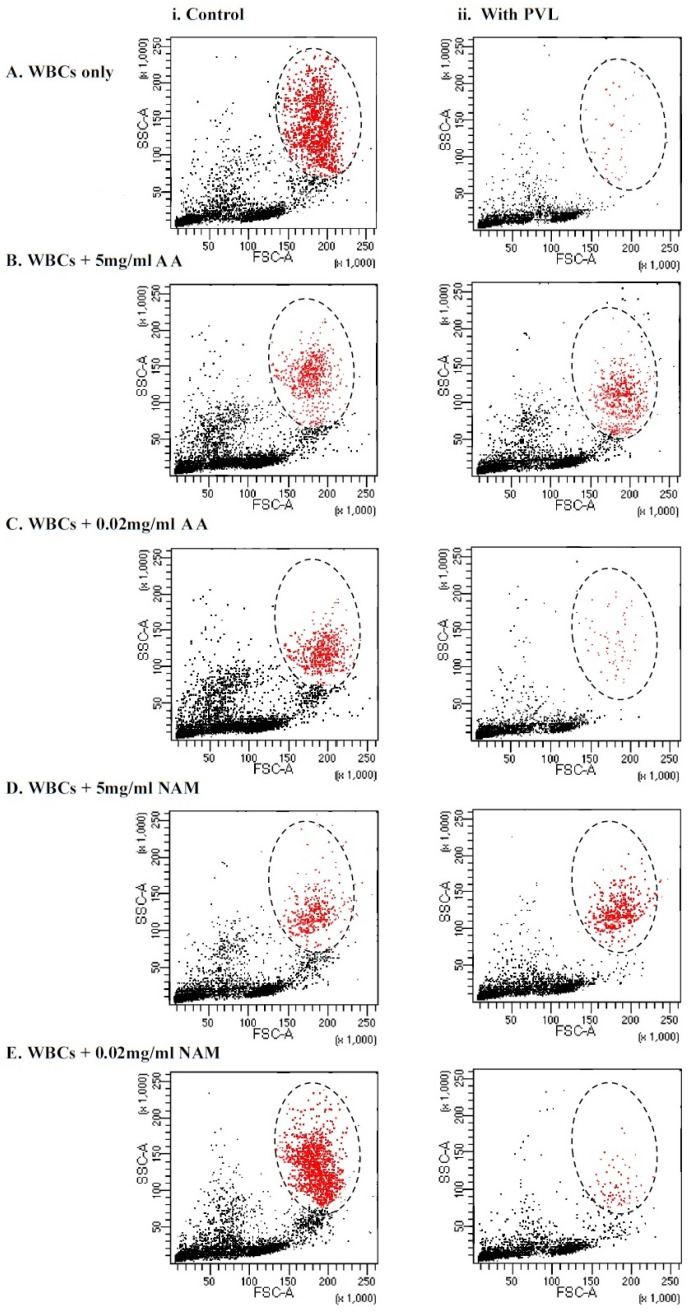
Immunophenotyping of tested WBCs based on forward (FSC) and side (SSC) scatter size distribution gating. Granulocytes are colored in red and the zone of selection is illustrated by an oval. (i) The assays on the left illustrate controls that did not undergo the toxin incubation. (ii) The assays on the right illustrate test experiments where the cells were treated with 1 µg/mL of PVL for a 2 h incubation period. (**A**) WBCs that were not incubated with antioxidants—0 mg/mL. (**B**) WBCs that were incubated with 5 mg/mL of VC. (**C**) WBCs that were incubated with 0.02 mg/mL of VC. (**D**) WBCs that were incubated with 5 mg/mL of NAM. (**E**) WBCs that were incubated with 0.02 mg/mL of NAM. WBCs = white blood cells, PVL = Panton–Valentine Leukocidin, AA = ascorbic acid, and NAM = nicotinamide.

In the current study, we tested whether antioxidant incubation would improve the viability of granulocytes after PVL treatment via flow cytometry size distribution, the dot plot shown in Figure 3. As shown in Table 1, no statistically significant difference in granulocytes viability after PVL treatment was demonstrated when incubated with 5 mg/mL of ascorbic acid and nicotinamide, whereas when using a low concentration of antioxidants (0.02 mg/mL), a significant difference was demonstrated (*p* < 0.001). This finding may illustrate that the cytotoxic effect of the toxin was neutralized by antioxidant incubation at a concentration of 5 mg/mL, making the difference in viability between the two assays statistically insignificant. Notably, incubating WBCs with higher concentrations (25 mg/mL and 10 mg/mL) of ascorbic acid and nicotinamide induced cell death without PVL challenge (data not shown).

Furthermore, a positive association between granulocyte viability and antioxidant incubation was determined, as granulocytes are three times more likely to be viable when incubated with 5 mg/mL of ascorbic acid and nicotinamide (OR 2.8 and 3, respectively, *p* < 0.01), as seen in Table 1. The cytoprotective mechanism of ascorbic acid and nicotinamide remains to be fully explained. However, many studies have illustrated this function in many cell types; for example, ascorbic acid contributed to neutrophil function recovery in furunculosis patients with defective neutrophil function [49]. In another report, ascorbic acid demonstrated amelioration of liver damage in the amoebic liver abscess (ALA) hamster model [50]. Similarly, vitamin B3 cytoprotective properties were illustrated in a stroke model study where nicotinamide ameliorated brain cell damage after focal ischemia-reperfusion [51]. Additionally, vitamin B3 increased cell viability in high glucose-treated corneal epithelial cells [52].

Unfortunately, the effect of antioxidants on the cytotoxicity of staphylococcal synergohymenotropic toxins, such as PVL, has not been investigated extensively in the literature, so we can only postulate the reason behind our findings. PVL pore-forming complex binds to C5aR1/2 on immune cells [11]. Through NLRP3 activation, the cytotoxic paradigm of PVL is suggested to be conducted [12]. Though PVL pathology and the exact cytotoxic molecular mechanism are not fully elucidated, in some instances, PVL is associated with ROS production that ultimately leads to DNA damage and cell death [53,54]. It is well-established that vitamin C and B3 have the ability to scavenge ROS and free radicals formed intracellularly [32,55]. For instance, oral and topical nicotinamide inhibited skin photocarcinogenesis that is mediated by ROS produced via UV radiations in murine models [56]. Additionally, ascorbic acid administration significantly reduced oxidative stress in the hippocampus stemming from lipid peroxidation and nitrite production in rat models after pilocarpine treatment [57]. Thus, neutralizing ROS produced from toxin treatment may have played a role in the demonstrated reduction of PVL cytotoxicity.

Furthermore, PVL cytotoxicity is associated with the release of proinflammatory cytokines, such as IL-1β, through the activation of NLRP3 inflammasome [3,12]. Many studies illustrated the inhibitory effect of vitamins B3 and C on certain components of the NLRP3 activation cascade or the mechanism overall. For instance, nicotinamide attenuated the activity of US Standard Endotoxin (EC-5), substantially reducing the production of the proinflammatory cytokines IL-1β, IL-6, and IL-8 in whole blood assays [58]. Additionally, vitamin B3 administration correlated with the downregulation of NLRP3, ASC, IL-6, and caspase-1 in diabetic mice models [45]. Similarly, ascorbic acid inhibited the inflammatory effect of lipopolysaccharide (LPS-G) antigen on human gingival mesenchymal stem cells (hGMSCs), subsequently reducing the expression of NLRP3, caspase-1, and IL-1β [59].

In conclusion, Panton–Valentine Leukocidin is a staphylococcal exotoxin that is associated with the severity of SSTIs, joint infections, and deep-seated abscesses, regardless of methicillin resistance. Liability of PVL-associated conditions transcended common risk factors of staphylococcal infections, affecting young immunocompetent individuals without underlying conditions.

Eliminating bacterial pathogens should not be the only aim in managing PVL-associated conditions. The removal of toxins, suppressing toxin production, and neutralizing the toxic effects of PVL are additional important objectives that may result in successful treatment [60]. In the current study, our findings illustrated that ascorbic acid and nicotinamide exhibit an inhibitory effect on PVL cytotoxicity and promote cell viability. Further investigations are needed to assess whether these antioxidants may be used as viable options in PVL cytotoxicity attenuation in PVL-associated diseases.

## 3. Materials and Methods

### 3.1. White Blood Cell Isolation

Peripheral blood was collected from 8 healthy donors via venipuncture in purple-capped EDTA K3 tubes (BIOTA-PCE3K8, Biota, Istanbul, Turkey). White blood cells were isolated through a gradient of Histopaque 1077 (Sigma-Aldrich, St. Louis, MO, USA), as recommended by the manufacturer’s datasheet, and pooled. Trypan blue exclusion microscopy (ab233465, Abcam, Hong Kong, China) was used to ascertain the viability of harvested WBCs (a range of 95–99%) before commencing the ‘assay procedure’. Harvested WBCs (1 × 10^6^ cells/mL) were incubated in RPMI 1640 (R0883-6X1L, Sigma-Aldrich, MO, USA) without additives and used immediately.

### 3.2. Panton–Valentine Leukocidin (PVL)

Purified subunits of PVL were purchased from Integrated BioTherapeutics, Rockville, MD, USA, LukS-PV (Cat#0530-001) and LukF-PV (Cat#0536-001). A 5 µg/mL PVL stock was prepared by combining both subunits in PBS + 15% Glycerol.

### 3.3. Antioxidant Preparation

Stocks (20 mg/mL) of ascorbic acid (#A92902, Sigma-Aldrich, MO, USA) and nicotinamide (#N3376, Sigma-Aldrich, MO, USA) were prepared in RPMI 1640 and pH was adjusted to neutral (pH = 7.2).

### 3.4. Assay Procedure

WBCs (1 × 10^6^ cells/mL) were incubated with a 2.5-fold serial dilution of ascorbic acid or nicotinamide (5, 2, 0.8, 0.32, 0.13, 0.05, and 0.02 mg/mL) in RPMI 1640 for 2 h at 37 °C and 5% CO_2_. Then, PVL (LuKS/F-PV) was added at a concentration of 0.5 µg/mL or 1 µg/mL to the WBCs and incubated for an additional 2 h at 37 °C and 5% CO_2_. Afterward, viability measurement and a cytotoxicity assay were conducted. Appropriate controls were used in each step.

Note: Determining the optimum assay procedure required a period of trials in the early stages of the study. Challenging WBCs with PVL (0.5 and 1 µg/mL) before, with, and after antioxidant treatment (starting concentrations of 25, 10, and 5 mg/mL) were attempted at 10 min, 30 min, 1 h, 2 h, and 3 h. At 10 min, no measurable changes occurred. Challenging the cells with PVL prior to antioxidant treatment (for 30 min and more) resulted in the release of LDH into the enclosed microenvironment, covering any potential ameliorating effect caused by the antioxidant (the OD was 2.5–3 at 650 nm). Likewise, applying the toxin with the antioxidant at the same time demonstrated the same observations. At 2 and 3 h incubations, the results were very similar, statistically showing no significant difference. All in all, we pursued the method that provided a clear, reliable, and repeatable association between the toxin and the antioxidant.

### 3.5. Cytotoxicity Assay

After the ‘assay procedure’ incubation periods, the cytotoxicity assay was performed in triplicates using the Cytotoxicity Detection Kit PLUS (LDH) (Cat#04744934001, Roche Diagnostics, Mannheim, Germany), according to the manufacturer’s manual. This assay is based on the measurement of lactate dehydrogenase (LDH) activity released from the damaged cell.

Three controls were included: the background control (media only), the low control (untreated cells), and the high control (cells treated with a lysis buffer for maximum LDH release).

To determine the experimental absorbance values, the average absorbance values of the triplicate samples and controls were calculated and subtracted from the absorbance values of the background control. The absorbance was measured via a FLUOstar^®^ OMEGA Plate Reader (BMG LABTECH, Ortenberg, Germany) at 490 nm, and the reference wavelength was 650 nm.

The cytotoxicity rate was determined using the following equation:

(1) $$\mathrm{Cytotoxicity}\;\left(\%\right)=\frac{\exp.\;\mathrm{value}-\mathrm{low}\;\mathrm{control}}{\mathrm{high}\;\mathrm{control}-\mathrm{low}\;\mathrm{control}}\times 100$$

### 3.6. Viability Measurement

After the ‘assay procedure’ incubation periods, WBCs were washed thrice and suspended in 1X PBS. A total of 10,000 events were gated and analyzed via a BD FACSCanto™ Flow Cytometer (Becton and Dickinson (BD), Franklin Lakes, NJ, USA) based on the forward scatter (FSC) and the side scatter (SSC) parameters. The viability of granulocytes was determined through size distribution (FSC X SSC). Viability was calculated by the following equation:

(2) $$\mathrm{Viability}\;\left(\%\right)=\frac{\mathrm{Number}\;\mathrm{of}\;\mathrm{viable}\;\mathrm{cells}}{\mathrm{Total}\;\mathrm{number}\;\mathrm{of}\;\mathrm{cells}}\times 100$$

### 3.7. Statistical Analysis

Calculations and statistical analysis (descriptive statistics, unpaired *t*-tests, and odds ratios) were conducted using Microsoft Excel 365. All experiments were performed in triplicates and the data are expressed as Mean ± SD whenever applicable. Differences between values were considered to be significant when the *p*-value was <0.001. The odds ratio was used to determine the efficacy of the antioxidant treatment for the viability of PVL-challenged cells.

The odds ratio calculation was performed according to the following formula:

(3) $$\mathrm{Odds}\;\mathrm{Ratio}\;\left(\mathrm{OR}\right)=\frac{a/b}{c/d}=\frac{a\;\times \;d}{c\;\times b}$$

where
Treatment statusPVL-challengedWithout PVLAntioxidant treatmentabWithout antioxidant treatmentcd

## Figures and Tables

**Table 1:** Granulocyte viability rates with or without PVL incubation were determined through forward (FSC) and side (SSC) scatter gating. Cells were incubated with 5, 0.02, and 0 mg/mL of AA or NAM. An Independent *t*-test was conducted to test the statistical significance of PVL treatment difference with the control (significant when *p* < 0.001).

Assay	Viable Granulocytes %		Viability Difference			Odds Ratio		
	Mean	SD	Δ*	Viability % **	*t*-Test (*p*-Value)	OR	95% CI	*p*-Value
Control (Cells only)	53	1.15	36	32	<0.001	-	-	-
Cells + PVL (1 µg/mL)	17	1.15						
Cells + AA (5 mg/mL)	49	1.15	5	90	0.002	2.8	1.4–5.5	0.003
Cells + AA (5 mg/mL) + PVL (1 µg/mL)	44	1						
Cells + AA (0.02 mg/mL)	46	0.6	27	41	<0.001	1.3	0.6–2.8	0.5
Cells + AA (0.02 mg/mL) + PVL (1 µg/mL)	19	1.5						
Cells + NAM (5 mg/mL)	44	1.5	2	95	0.049	3	1.5–5.9	0.002
Cells + NAM (5 mg/mL) + PVL (1 µg/mL)	42	1.5						
Cells + NAM (0.02 mg/mL)	56	0.5	34	39	<0.001	1.2	0.59–2.6	0.6
Cells + NAM (0.02 mg/mL) + PVL (1 µg/mL)	22	1.15						

* Δ (difference) = Control (PVL treatment) values. ** Viability % = $\left(1-\frac{\Delta }{\mathrm{control}}\;\right)\times 100$. NAM = nicotinamide, PVL = Panton–Valentine Leukocidin, and AA = ascorbic acid.

## Data Availability

Not applicable.

## Supplementary Materials

The following supporting information can be downloaded at https://www.mdpi.com/article/10.3390/toxins15010038/s1, Table S1: PVL (0.5 µg/mL) cytotoxicity rates determined through LDH release, Table S2: PVL (1 µg/mL) cytotoxicity rates determined through LDH release.
