# Supplementary material for: The Effect of Ascorbic Acid and Nicotinamide on Panton–Valentine Leukocidin Cytotoxicity: An Ex Vivo Study
Source: Toxins (Basel). 2023 Jan 4;15(1):38. doi: 10.3390/toxins15010038 (PMC9865643; doi:10.3390/toxins15010038)
Supplement: Supplementary file 1 [file toxins-15-00038-s001.zip › toxins-2113448-supplementary.pdf]

Table S1. PVL (conc. 0.5μg/ml) cytotoxicity rates determined through LDH release. Cells were treated with serial dilutions of AA and NAM individually. T-test was conducted to determine the statistical significance of antioxidant treatment when compared with the control (Cells + PVL only), significance = $p < 0.001$.

Assay	Cytotoxicity%		t-test
(PVL 0.5μg/ml)	Mean	SD	P-value
Control (Cells + PVL only)	30	0.5	-
Cells + VC 5mg/ml + PVL	10	1	<0.001
Cells + VC 2mg/ml + PVL	14	1	<0.001
Cells + VC 0.8mg/ml + PVL	16	0.6	<0.001
Cells + VC 0.32mg/ml + PVL	21	1.2	<0.001
Cells + VC 0.13mg/ml + PVL	21	1.2	<0.001
Cells + VC 0.05mg/ml + PVL	23	0.6	<0.001
Cells + VC 0.02mg/ml + PVL	29	0	0.03
Cells + NAM 5mg/ml + PVL	8	0.6	<0.001
Cells + NAM 2mg/ml + PVL	10	1	<0.001
Cells + NAM 0.8mg/ml + PVL	14	1.2	<0.001
Cells + NAM 0.32mg/ml + PVL	15	1	<0.001
Cells + NAM 0.13mg/ml + PVL	16	1.2	<0.001
Cells + NAM 0.05mg/ml + PVL	22	0.8	<0.001
Cells + NAM 0.02mg/ml + PVL	29	1.2	0.5

Table S2. PVL (conc. 1µg/ml) cytotoxicity rates determined through LDH release. Cells were treated with serial dilutions of AA and NAM individually. T-test was conducted to determine the statistical significance of antioxidant treatment when compared with the control (Cells + PVL only), significance = $p < 0.001$.

Assay (PVL 1µg/ml)	Cytotoxicity%		t-test
	Mean	SD	
Control (Cells + PVL only)	45	0	-
Cells + VC 5mg/ml + PVL	17	1	<0.001
Cells + VC 2mg/ml + PVL	26	1.2	<0.001
Cells + VC 0.8mg/ml + PVL	28	1	<0.001
Cells + VC 0.32mg/ml + PVL	30	0.6	<0.001
Cells + VC 0.13mg/ml + PVL	32	0.6	<0.001
Cells + VC 0.05mg/ml + PVL	38	0.9	<0.001
Cells + VC 0.02mg/ml + PVL	42	0.6	<0.001
Cells + NAM 5mg/ml + PVL	11	0.6	<0.001
Cells + NAM 2mg/ml + PVL	14	0.6	<0.001
Cells + NAM 0.8mg/ml + PVL	16	1	<0.001
Cells + NAM 0.32mg/ml + PVL	18	1	<0.001
Cells + NAM 0.13mg/ml + PVL	28	1.2	<0.001
Cells + NAM 0.05mg/ml + PVL	35	1	<0.001
Cells + NAM 0.02mg/ml + PVL	40	0.3	<0.001
